# Signalling by Transforming Growth Factor Beta Isoforms in Wound Healing and Tissue Regeneration

**DOI:** 10.3390/jdb4020021

**Published:** 2016-06-22

**Authors:** Richard W.D. Gilbert, Matthew K. Vickaryous, Alicia M. Viloria-Petit

**Affiliations:** 1 School of Medicine, University College Cork, Cork, Ireland; Richard.wd.gilbert@gmail.com; 2 Department of Biomedical Sciences, Ontario Veterinary College, University of Guelph, Guelph, ON N1G 2W1, Canada

**Keywords:** transforming growth factor beta, TGFβ, isoforms, tissue regeneration, wound healing

## Abstract

Transforming growth factor beta (TGFβ) signalling is essential for wound healing, including both non-specific scar formation and tissue-specific regeneration. Specific TGFβ isoforms and downstream mediators of canonical and non-canonical signalling play different roles in each of these processes. Here we review the role of TGFβ signalling during tissue repair, with a particular focus on the prototypic isoforms TGFβ1, TGFβ2, and TGFβ3. We begin by introducing TGFβ signalling and then discuss the role of these growth factors and their key downstream signalling mediators in determining the balance between scar formation and tissue regeneration. Next we discuss examples of the pleiotropic roles of TGFβ ligands during cutaneous wound healing and blastema-mediated regeneration, and how inhibition of the canonical signalling pathway (using small molecule inhibitors) blocks regeneration. Finally, we review various TGFβ-targeting therapeutic strategies that hold promise for enhancing tissue repair.

## 1. Transforming Growth Factor Beta (TGFβ) Signalling

The TGFβ superfamily consists of 33 members, most of which are dimeric, secreted polypeptides. In addition to the three prototypic TGFβ isoforms (TGFβ1, TGFβ2 and TGFβ3), the superfamily also includes the activins, inhibins, **B**one **M**orphogenic **P**roteins (BMPs), **G**rowth and **D**ifferentiation **F**actors (GDFs), myostatin, nodal, leftys and **M**ullerian **I**nhibiting **S**ubstance (MIS) [1]. The scope of this review will be largely limited to the three isoforms of TGFβ ligand: TGFβ1, TGFβ2 and TGFβ3. The specific roles of other members of this superfamily in tissue repair and regeneration have been thoroughly reviewed elsewhere (see [2] and [3] for activins and BMPs, respectively).

Members of the TGFβ family are widely recognized as key signal transducers among multicellular animals (metazoans), including both invertebrates (e.g., the placozoan *Trichoplax adhaerens* [4], and acorn worms [5]), and vertebrates. The three prototypic TGFβ isoforms, TGFβ1, TGFβ2 and TGFβ3, are structurally similar cytokines encoded by separate genes that act in autocrine and paracrine manners to regulate early embryonic development, the maintenance and regeneration of adult tissues, as well as various disease processes [6,7,8]. TGFβ ligands are secreted as inactive precursors bound to latency-associated peptides and are either directly activated or embedded in the extracellular matrix (ECM) to be activated at a later time. In most tissues, significant amounts of TGFβ are stored in the ECM [9]. TGFβ ligand activation is accomplished by the lytic action of proteases including elastase and **m**atrix **m**etallo**p**roteases (MMPs), or through conformational changes induced by various integrins [10,11].

Following release, TGFβ ligands evoke their cellular effects on target cells by binding to transmembrane dual specificity receptors, which possess strong serine/threonine kinase activity and weak tyrosine kinase activity [12,13]. TGFβ receptors are the sole cell surface serine/threonine kinase receptors known in humans [14], and can be divided into three classes: type I (TβRI; also known as **a**ctivin-**l**ike **k**inase, TβRI/ALK), type II (TβRII), and type III (TβRIII). To activate cellular signalling, the ligand first binds to a dimer of constitutively active TβRII, which is then brought into close proximity with a dimer of TβRI (ALK5 in the majority of cell types; ALK5 or ALK1 in endothelial cells [15]), allowing TβRII to phosphorylate TβRI [12,16]. Once activated, the tetrameric receptor complex initiates an intracellular cascade that evokes the activation of canonical and non-canonical signalling pathways. Type III receptors, including the co-receptors endoglin and betaglycan, mediate the binding of specific TGFβ isoforms and further regulate receptor activity [6]. 

Endoglin binds to TβRII-associated TGFβ, but not to free TGFβ, and is best known from its role in angiogenesis [1,17]. Endoglin expression by endothelial cells enhances TGFβ signalling via ALK1-Smad1 and inhibits signalling via ALK5-Smad3. However, it is important to note that endoglin function is multifaceted: it exists in two different splice variants that have opposing functions, and it can serve as a co-receptor for other TGFβ family ligands, including BMP9 and BMP10 [18]. In addition to its role in angiogenesis, emerging data indicates that endoglin is also involved (in a context-dependent manner) in fibrosis and scleroderma [18]. Similar to endoglin, betaglycan is a TβRIII with multiple functions. These include ligand presentation to the type II receptor, and enhancement or inhibition of the action of ligands in a context-dependent manner (reviewed in [19])

Canonical TGFβ signalling pathway is mediated through cytoplasmic proteins known as the SMADs (**s**mall **m**others **a**gainst **d**ecapentaplegic) [20]. SMAD proteins contain two globular domains, termed MH1 and MH2, connected by a linker domain. The MH1 domain contains a DNA-binding domain, while the MH2 domain contains a series of hydrophobic patches that facilitate protein-protein interactions [20]. In vertebrates, there are eight members of the SMAD family, SMADs 1−8. SMADs are categorized into three classes depending on their structure and function. Receptor activated or R-SMADs (SMADs 1−3,5,8) interact with activated TβRI, resulting in their C-terminal phosphorylation [20,21]. In most cases, TGFβ’s (as well as activin, myostatin and nodal ligands) activation of TβRI results in the C-terminal phosphorylation of SMAD2 and SMAD3, whereas BMPs and GDFs cause the C-terminal phosphorylation of SMAD1, SMAD5 and SMAD8 [20,21]. Similarly, TGFβ-dependent activation of ALK1 on endothelial cells, which primarily occurs in response to low ligand concentration, also results in activation of SMAD1/5 [15].

An important mediator of SMAD2/3 activation is the adaptor protein known as **S**mad **a**nchor for **r**eceptor **a**ctivation (SARA) [22]. SARA interacts with both the plasma membrane and SMAD2/3 (the latter via SMAD’s MH2 domain); this ensures SMAD’s proximity to the plasma membrane and the TβR complex, thus facilitating activation of SMAD [22]. SARA’s key role in TGFβ signalling is not limited to SMAD activation; SARA may also modulate the outcome and duration of the signal by regulating the balance between SMAD2 and SMAD3 and facilitating SMAD7-mediated TβRI dephoshorylation [23]. However, the extent of SARA’s involvement in TGFβ signalling might be cell-type dependent, as it was recently observed that in Hela and B-cell lymphoma cells, SARA levels do not necessarily correlate with SMAD activation, nuclear translocation and SMAD-dependent gene expression [24]. 

Upon C-terminal phosphorylation the common-mediator SMAD, (SMAD4) interacts with activated R-SMAD complexes to assist with their nuclear translocation. The nuclear-cytoplasmic shuttling of SMAD proteins plays a substantial role in modulating TGFβ signalling, and is determined by different mechanisms for individual SMADs. Numerous proteins have been demonstrated to play essential roles in this shuttling, including components of the nuclear pore complex, importins, exportins, and mediators of the Hippo signalling pathway [12,25].

Following nuclear translocation, activated SMAD transcriptional complexes bind to target DNA sequences to activate or repress gene expression. The recognition of specific DNA sequences and their ability to activate or repress gene expression is determined by the isoform of SMAD that is present, as well as numerous protein-protein interactions with transcriptional co-activators and co-repressors mediated through SMAD’s MH2 domain [4]. This ability of SMAD proteins to interact with numerous other proteins allows them to act as an integration hub for cell signalling crosstalk and greatly influences signalling outcome [4].

The extent of SMAD signalling activation is modulated by different mechanisms, including competitive receptor binding by R-SMADs and I-SMADs, the specific and timely degradation of signalling mediators, and receptor trafficking. I-SMADs (SMADs 6 and 7) bind directly to the TβR complex and block R-SMAD access to the receptor [26]. I-SMADs also compete with R-SMADs in the nucleus. Via its MSH2 domain, SMAD7 binds directly to DNA and prevents SMAD2, 3 and 4 from binding [1]. Ubiquitin-dependent proteasomal degradation of TGFβ-activated R-SMADs (such as SMADs 1, 2 and 3) is mediated by different E3-type ubiquitin ligases, and modulates both their steady-state levels, as well as the duration of their activated state. Among these, the best-documented are the **S**mad **u**biquitination **r**elated **f**actors 1 and 2 (Smurf1 and 2, respectively) [26]. SMAD4 is also targeted for proteasome-dependent degradation by a Smurf-independent pathway that might involve the SCFSkp2 complex [27]. Finally, I-SMADs also serve as adaptors that recruit the E3 ubiquitin ligases Smurf 1 and 2 to the TβR complex, facilitating proteasome-dependent degradation of activated TβRI [26]. SMAD7 can additionally recruit the phosphatase GADD34-PP1c to the activated TβR complex to attenuate signalling [1].

The localization of the TβR complex to specific membrane domains is key for signalling modulation, as it dictates its internalization via different routes and determines whether or not signalling will occur [28]. Internalization of the receptor via **c**lathrin-**c**oated **v**esicles (CCV) into **e**arly **e**ndosome **a**ntigen 1 (EEA-1)-postive and SARA-containing endosomes promotes signalling [29]. In contrast, internalization via membrane rafts (membrane domains of tightly packed cholesterol-sphingolipids protein complexes) into caveolin-positive vesicles results in receptor degradation and prevents signalling [29]. The latter vesicles specifically carry the inhibitory SMAD7, which by associating with Smurf2 facilitates Smurf-mediated targeting of TβRI for proteasome-dependent degradation [29]. Although what causes the receptors to segregate into these two different routes is not fully understood (ligand binding does not necessarily favors one route over the other [26]), it is known that the extracellular domain of TβRII (possibly via interaction with other cell surface glycoproteins) is required for partitioning into membrane rafts [30]. Further, when TβRIII/betaglycan is present, it recruits both TβRII and TβRI to non-raft membrane fractions, thus promoting SMAD signalling [31]. Taken together, these results indicate that the levels of expression of the TGFβ receptors themselves, and in particular betaglycan, dictate the extent of canonical signalling activation via modulation of receptor trafficking.

Although the three TGFβ isoforms primarily signal through the canonical SMAD2/3 pathway, numerous non-SMAD signalling pathways (referred to as non-canonical) are also activated by TGFβ ligands. These pathways include the Ras/MAPK/Erk pathway [13] the PI3K/Akt pathway [32], the TAK1-p38/JNK pathway [33], and the Par6-Polarity pathway [34]. Previous studies indicate important roles of non-canonical signalling in determining the functional outcome of TGFβ [35], including tissue repair [36,37].

Despite sharing 71%–76% sequence identity and signalling through the same canonical SMAD intermediates (SMAD2 and SMAD3), a growing body of evidence suggests that the three TGFβ isoforms have different physiological roles [38]. Each TGFβ isoform is transcribed from a unique promoter and has a distinct pattern of tissue expression [39]. The differences in isoform expression patterns are reinforced by non-overlapping phenotypes seen in TGFβ isoform-specific transgenic and knockout mice [38]. Some of the most well-studied examples of TGFβ isoform-specific biology are cardiac development [40], palate formation [41], and cutaneous wound healing; the latter will be discussed in Section 2.

Overall, the outcome of TGFβ signalling input is highly context-dependent, as it is the net result of numerous contributing factors, including: the specific ligand(s) present in the microenvironment; the bioavailability and concentration of such ligands; the cell type; the levels of signalling mediators within the cell; the extent of activation of canonical *versus* non-canonical signalling pathways; and the extent to which both of these branches of TGFβ signalling crosstalk with signalling inputs via other receptor systems, both in the cytoplasm and in the nucleus [4]. Importantly, increasing evidence indicates that major modifying signalling inputs are mediated by the cellular cytoskeleton in response to mechanical stimuli, such as loss of integrity of cell-cell contacts [42], cellular tension [43], and ECM stiffness [44,45]. Mechanotransduction of these stimuli in the presence of active TGFβ signalling results in synergistic responses between mechanosensitive transcriptional co-activators and TGFβ-regulated signal transducers, such as the R-SMADs [46,47,48]. As discussed in more detail below, this synergy plays an important role during key steps of wound healing and regeneration, such as fibrogenesis [46,47].

## 2. Cutaneous Wound Repair

Among vertebrates, the reparative response to injury follows a stereotypical sequence of events that can be divided into three main overlapping phases: hemostasis and inflammation; proliferation; and maturation and remodeling [49,50]. Throughout these events, TGFβ plays a number of crucial roles that vary in a context and cell type-dependent manner. The pleiotropic effects of TGFβ include regulating cell proliferation, differentiation, migration, invasion and chemotaxis of the epithelial, fibroblastic and immune cell tissue compartments (the latter involved in inflammatory response), as well as endothelial cell proliferation, migration and invasion, and mural cell maturation (to generate functional blood vessels) during angiogenesis [1,51].

### 2.1. Hemostasis and Inflammation Phase

TGFβ isoforms demonstrate a number of dynamic interactions throughout the processes of hemostasis and inflammation. Following tissue injury, blood vessels rupture and the resulting exposure of platelets (thrombocytes) to sub-endothelial collagen causes platelet aggregation, degranulation and activation of the coagulation cascade [49]. Platelet alpha-granules are a particularly rich source of TGFβ1 (upwards of 40 to 100 times more than in other cell types) [52]. Alpha-granules also contain other TGFβ isoforms, although the ratio is heavily skewed (4000 TGFβ1: 1 TGFβ2: 10 TGFβ3) [53,54]. Platelet-induced activation of the coagulation cascade results in the formation of a fibrin clot which achieves hemostasis as well as serves as a scaffolding for the migration of inflammatory cells into the wounded tissue [49]. 

Following hemostasis, TGFβ next participates as a potent chemoattractant and inflammatory mediator for various types of immune cells, including neutrophils and other **p**oly**m**orpho**n**uclear (PMN) cells (basophils, eosinophils, mast cells; beginning 24 to 48 h after wounding) [55,56,57] and circulating monocytes (48 to 96 h post-wounding) [58,59,60]. Curiously, TGFβ ligands are also known to antagonize other neutrophil chemoattractants, such as interleukin-8, and can suppress the ability of immune cells to transmigrate into injured tissues [56,61]. Hence, TGFβ participates in both stimulating the initial immune response, through the recruitment of PMN, and limiting the extent of the inflammatory response [56]. Whereas platelets are characterized as being rich in TGFβ1, in neutrophils, the ratio of TGFβ isoforms is biased towards TGFβ3 (12 TGFβ1: 1 TGFβ2: 34 TGFβ3), indicating the possibility of isoform-specific differences throughout the wound-healing process [54]. Following their recruitment, many subsequent roles of macrophages—including the initiation of granulation tissue formation and angiogenesis─are also known to be mediated by TGFβ [50,58]. 

### 2.2. Proliferative Phase

The proliferative phase involves three major TGFβ-mediated events: re-epithelialization; angiogenesis; and extracellular matrix (ECM) synthesis. In response to injury, epithelial cells located at the wound margins become activated and undergo a phenotypic change characterized by an alteration of their cytoskeleton and the dissolution of cell-cell contacts [62,63]. Migration and proliferation of epithelial cells is driven by a variety of autocrine and paracrine signalling pathways (reviewed by [63] and [64]), of which TGFβ is one of the most extensively studied. Prior to injury, TGFβ1 in the epidermis functions as a homeostatic cytokine, blocking cell-cycle progression and suppressing epithelial hyperplasia [65,66,67]. Following injury, all three TGFβ isoforms promote re-epithelialization [67,68,69], and their abolishment (with the use of neutralizing antibodies) impairs wound closure [69,70,71,72]. However, whereas TGFβ1 acts to promote keratinocyte migration *in vitro* [67], TGFβ3 does not [69].

The key mechanism involved in re-epithelialization is the **e**pithelial to **m**esenchymal **t**ransition (EMT) [73]. Key cellular events during EMT, including the loss of cell-cell contacts and increased motility, are driven by both canonical and non-canonical TGFβ signalling [73]. Changes in the levels of SMAD3 might play an important role in the switch of TGFβ function from a growth-suppressing cytokine in intact epithelium to an EMT-promoting one in wounded epithelium. SMAD3 mediates TGFβ’s growth-suppressive effects, and a decline in endogenous SMAD3 occurs in parallel to EMT and leads to loss of growth-inhibitory response to TGFβ during this process [74]. In agreement with these findings, mice that are heterozygous or null for SMAD3 show enhanced re-epithelialization and wound closure [75,76].

Epithelial cell injuries, such as those involving disruption of the Crumbs complex that associates with the tight junction (apical cell-cell contacts), are also known to sensitize cells to TGFβ-mediated EMT by enhancing nuclear translocation of SMAD2/3 via the Hippo pathway mediators TAZ (**t**ranscriptional co-**a**ctivator with PD**Z**-binding domain) and YAP (**Y**es-**a**ssociated **p**rotein) [46,77]. Interestingly, TAZ silencing prevents robust expression of alpha **s**mooth **m**uscle **a**ctin (αSMA) by TGFβ and subsequent epithelial to myofibroblast conversion in wounded epithelium [46], and skin-specific deletion of both TAZ and YAP in adult mice impairs skin regeneration after wounding [78]. This impairment was in part attributed to the role of TAZ/YAP in maintaining the stem-cell population of the basal layer of the skin [78]. Together, these observations suggest that a TGFβ and Hippo signalling crosstalk mediates TGFβ’s wound-healing properties.

Another key event during the proliferation phase is angiogenesis. Angiogenesis involves the invasion of the wound bed by capillary sprouts to create a *de novo* microvascular network [79,80,81,82]. Although still not fully understood, due to its context-dependency, a role for TGFβ as a modulator of angiogenesis has long been recognized [83]. TGFβ’s ability to induce angiogenesis might be linked, at least in part, to its capacity to promote **v**ascular **e**ndothelial **g**rowth **f**actor (VEGF) expression at the site of injury. VEGF mediates angiogenic activity during the proliferative phase of wound healing [80], and TGFβ is known to recruit VEGF-producing hematopoietic effector cells to promote angiogenesis *in vivo* [84]. All three TGFβ isoforms can also induce **endo**thelial to **m**esenchymal **t**ransition (EndoMT) [40], which has been widely implicated in pathologic fibrosis of various organs (including the skin [85,86]), as well as the sprouting phase of angiogenesis [87]. 

Finally, TGFβ is involved in ECM synthesis and the recruitment of fibroblasts from the adjacent dermis [88], as well as from perivascular sources (e.g., pericytes) and bone marrow (*i.e.*, fibrocytes) [89,90,91]. Once they have entered the wound bed, fibroblasts proliferate and begin synthesizing the provisional ECM (mostly collagen and fibronectin) that precedes the formation of granulation tissue proper. Granulation tissue is a transient, heavily vascularized reparative organ characterized by a loose matrix of collagen, fibronectin and hyaluronic acid interspersed with fibroblasts and macrophages [49,50]. TGFβ ligands play a fundamental role in fibroblast regulation and the production of granulation tissue. TGFβ1 mediates fibroblast collagen production (specifically type I and III), as well as in the inhibition of MMPs [92]. Related to this, TGFβ1-mediated signalling has been implicated in diseases characterized by excessive collagen deposition including keloids and scleroderma [92,93,94]. Importantly, while TGFβ1 and TGFβ2 promote collagen deposition and scar formation, TGFβ3 appears to be anti-fibrotic [95,96]. Hence, the combined effect of TGFβ3 and TGFβ1 is interpreted as a fine-tuning of collagen production [92,97]. As the proliferative phase of wound healing progresses, a subset of fibroblasts will differentiate into myofibroblasts and another subset will undergo apoptosis, thereby marking the beginning to the final stage of wound healing, the remodeling phase [49]. 

### 2.3. Remodeling Phase

The final phase of wound healing is remodeling, involving the apoptosis of resident cells (including fibroblasts and endothelial cells), as well as wound contracture, and the replacement of fibronectin and type III collagen in the wound bed with type I collagen [49,92]. As a result, the once highly cellular and heavily vascularized mass of granulation tissue is transformed into a largely avascular and acellular scar [88,91]. Wound contracture is facilitated by myofibroblasts, a population of fibroblasts that acquire a contractile phenotype, as evidenced by their expression of αSMA [91]. The acquisition of αSMA expression is controlled by TGFβ1, through SMAD-dependent and independent transcriptional activity at the αSMA promoter [44,91,98], as well as by mechanical loading of the wound environment [91]. Curiously, myofibroblasts are absent from the wound bed during the earlier phases of wound healing when levels of TGFβ1 are at their highest [91]. One explanation is that in order to express αSMA, fibroblasts require a combination of a stiff milieu/mechanical stress and TGFβ1 [91,98]. In support of this prediction, *in vitro* experiments have demonstrated that even in the presence of adequate TGFβ1 levels, fibroblasts fail to transition to myofibroblasts if plated on low stiffness environments [44]. This might be related to the observation that a mechanoresistant/stiff ECM facilitates the activation of latent, ECM-sequestered TGFβ1 by the myofibroblasts themselves [45]. In this study, a stiff ECM was found to be required for integrin-mediated activation of self-produced TGFβ1 by myofibroblast, as a result of their cytoskeletal contraction caused by ECM tension [45]. In agreement with these findings, myofibroblast-populated wounds displayed a higher level of SMAD2/3 activation in stressed as compared to relaxed tissue, despite similar levels of TGFβ1 and TβRII [45]. This suggests that during wound remodeling, TGFβ1 activation (and the consequent maintenance of the myofibroblast phenotype) is restricted to areas with a stiff ECM, equivalent to that encountered in the late-wound granulation tissue [45]. 

Although the mechanisms through which fibroblasts and myofibroblasts interpret their environment are not completely understood, members of the Hippo signalling pathway, such as TAZ, are likely involved in mechano-sensing the tissue environment and modulating TGFβ1 responsiveness [46,48]. In agreement with this notion, TAZ was shown to confer SMAD3 sensitivity to the αSMA promoter, and to facilitate αSMA expression in response to TGFβ1 in combination with mechanical stretch [47]. In contrast, when there was only mechanical stretch (but no TGFβ1), another major mechanosensitive transcriptional co-activator known as **m**yocardin **r**elated **t**ranscription **f**actor (MRTF), interacted with TAZ and SMAD3 to suppress SMAD3-TAZ-mediated activation of the αSMA promoter [47]. Together, these findings support a model whereby stretch alone promotes a limited contractile response, possibly promoting healing, while stretch plus TGFβ1 favors the formation of fibrotic tissue [47].

Similar to TGFβ1, TGFβ2 is also a potent inducer of the fibroblasts to myofibroblast transition (both *in vitro* and *in vivo*) [99]. In contrast, the role of TGFβ3 is more complex. While TGFβ3 appears to promote the acquisition of a myofibroblast phenotype *in vitro*, *in vivo* it inhibits myofibroblast formation [96,99]. 

### 2.4. Exceptions to Scar Formation in Mammals

Among mammals it is well understood that most injuries to the skin are resolved with the formation of scar tissue. Although scar tissue acts to help restore structural integrity and homeostasis, it is a dysfunctional replacement. Conspicuously, scar tissue fails to re-develop hair follicles and glands, as well as the protein elastin and the original basket-weave collagen architecture of the dermis. As a result, scars lack the tensile strength of uninjured skin [96,100]. However, a number of remarkable exceptions to this mammalian scarring paradigm exist. For example, in some species of African spiny mice (*Acomys*), large sections of dorsal body skin can be shed (autotomized) and then regenerated scar-free, complete with hair follicles and glands [101]. These species can also regenerate through-and-through ear punch wounds, regenerating skin and cartilage [101]. Curiously, a recent qRT-PCR screen has revealed that *TGF*β*1*, typically considered a pro-inflammatory cytokine, is significantly upregulated during wound healing in *Acomys*: a seven-fold increase compared to uninjured skin; in mice (which scar) the increase is only three-fold [102]. 

Another notable example comes from fetal mammals. Many mammals (including humans, rats, mice, pigs and monkeys) are capable of scar-free cutaneous healing in the early- to mid-gestation stages of fetal development [88,103,104]. Although details of the mechanisms permitting scar-free fetal wound healing remain to be fully elucidated, a role for TGFβ has been established [88]. One of the key observations is that the expression of TGFβ isoforms differs between the fetal and adult responses to injury. More specifically, whereas adult cutaneous wounds demonstrate high levels of TGFβ1 and TGFβ2, but low levels of TGFβ3, the expression pattern in the fetal wound is the reverse (high expression of TGFβ3, low expression of TGFβ1 and TGFβ2) [105,106]. If fetal wounds are treated with exogenous TGFβ1, the result is scarification [107]. Alternatively, if adult wounds are treated with exogenous TGFβ3, or if endogenous TGFβ1 and TGFβ2 are blocked (e.g., with neutralizing antibodies), the severity of scarring is reduced [96]. These observations combined with numerous other examples from adult wound healing place TGFβ isoforms, and in particular their relative ratios, as a driving force in determining the balance between tissue repair and tissue regeneration. To better understand this phenomenon, the next section examines the role of TGFβ isoforms in species that possess the unique ability, like fetal wounds, to heal without scarification.

**Figure 1:**
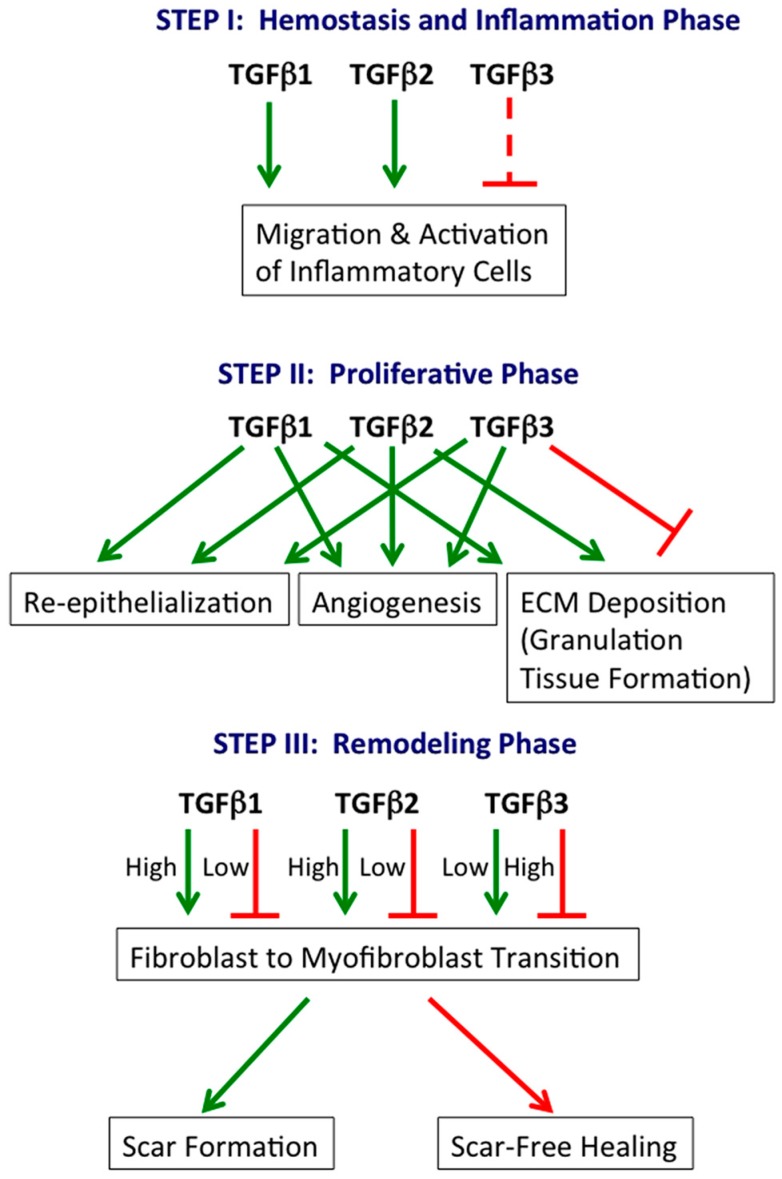
TGFβ isoforms in cutaneous wound healing. TGFβ1, TGFβ2 and TGFβ3 play central roles in all three phases of wound healing. Generally, TGFβ1 and TGFβ2 are stimulatory, while TGFβ3 is inhibitory. However, TGFβ3 can also stimulate specific processes (e.g., re-epithelialization). Green arrow: stimulatory; continuous red line: inhibitory; dashed red line: potentially inhibitory, inferred from relative levels at the beginning (low) and end (high) of the hemostasis and inflammation phase.

The involvement of specific TGFβ isoforms in the three phases of cutaneous wound healing is summarized in Figure 1. 

## 3. Multi-Tissue Regeneration

### 3.1. Blastema Formation

Amongst vertebrates, many of the most striking examples of multi-tissue regeneration begin with the formation of a mass of mesenchymal-like cells at the wound site—the blastema [108]. Although the blastema appears to be composed of a homogeneous population of undifferentiated cells, various recent studies have demonstrated that blastema cells are actually a heterogeneous pool of lineage-restricted progenitor cells [109,110,111]. Consequently, blastema cells are not a pluripotent (or perhaps even multipotent) population, but instead retain a memory of their germ-layer origin (axolotls: [109], mouse digits: [111]). Details of blastema formation remain poorly understood, but it is predicted to be the result of either reprogramming events occurring amongst the different lineage restricted cell populations, or rapid expansion of tissue-specific stem-cell populations, or a combination of both [109,110,111].

One of the earliest signs of blastema-mediated (*i.e.*, epimorphic) regeneration is the formation of a wound epithelium. The wound epithelium first forms as original epidermal cells surrounding the wound migrate across the site of injury [112]. Once re-epithelialization is complete, the wound epithelium begins to thicken, resulting in a capping structure that closely resembles the **a**pical **e**ctodermal **r**idge (AER) observed during limb development [113,114]. In addition to thickness, the wound epithelium also differs from the pre-wounding epidermis in that it lacks the distinctive stratified appearance, basal keratinocyte polarity and a mature basal lamina [115,116]. Furthermore, the wound epithelium demonstrates unique protein and gene expression profiles compared to normal epithelium [117,118,119]. Independent reports have established that the wound epithelium is key for blastema induction and proliferation [114,120].

### 3.2. TGFβ in Multi-Tissue Regeneration

One of the best-documented investigative approaches to demonstrate the requirement for TGFβ signalling during *in vivo* regeneration involves the use of the potent small molecule inhibitor SB-431542. This is a selective inhibitor of the type I receptors ALK4, ALK5 and ALK7, and acts to inhibit phosphorylation of SMAD2 and SMAD3 [121]. In axolotls, TGFβ1 mRNA is normally upregulated by blastema cells during the early (preparatory) phase of limb regeneration [122]. Moreover, if amputated animals are treated with SB-431542, cell proliferation is halted, the blastema fails to form, and regeneration is prevented [122]. Similarly, spontaneous tail regeneration by *Xenopus* tadpoles involves an increase in phosphorylated SMAD2 (pSMAD2) expression, as well as an upregulation of TGFβ family members xTGFβ2 (similar to TGFβ2), xTGFβ5 (similar to TGFβ1), as well as xGDF11 and xActivin-βA [123]. When amputated tadpoles are treated with SB-431542, wound healing is blocked, cell proliferation is reduced, and the blastema fails to form [123]. 

Other evidence supporting the involvement of TGFβ in regeneration comes from experiments with zebrafish. Following tail fin amputation, spontaneous regeneration of the appendage involves a significant upregulation of activin-βA, one of the subunits of the activin complexes AB and B [124]. Treatment with SB-431542 results in an abnormal wound epidermis, reduced cell proliferation, and the failure of the blastema to properly form. To expand these findings, the authors then used knockdown morpholinos to silence activin-βA expression. The result was a 50% reduction in regenerated tail size [124]. Combined, these experimental observations support the role of TGFβ signalling in cell proliferation, in addition to blastema formation and maintenance. 

TGFβ signalling is also involved in zebrafish cardiac regeneration following cryoinjury. The cryoinjury method results in localized cell death along the ventricular wall, and has the advantage of histologically mimicking a myocardial infarction otherwise characteristic of mammals, including humans [125]. Myocardial repair is a two-step process, beginning with scar formation, which is then gradually replaced with new cardiac muscle [125]. During myocardial repair, all three TGFβ isoforms (TGFβ1, TGFβ2, TGFβ3), as well as activin βB (but not activin βA) were upregulated [126]. This increase in TGFβ ligands corresponds to a robust induction of pSMAD3 in both the injured myocardium and the uninjured myocardium directly adjacent to the wound, confirming activation of the TGFβ signalling pathway [126]. When cryoinjured fish were treated with SB-431542, myocardial regeneration failed. This regenerative failure is the result of both a suppression of initial collagen synthesis, thus limiting the early formation of a scar, combined with the inhibition of cardiomyocyte proliferation [126].

A possible role for activin-βA during regeneration has also been proposed for the leopard gecko following tail loss. Similar to *Xenopus* tadpoles, cells of the leopard gecko’s wound epithelium and blastema demonstrate widespread expression of pSMAD2 [127]. In order to identify the ligand(s) responsible for SMAD activation, a qRT-PCR screen was performed (including TGFβ1, TGFβ2, TGFβ3, and activin-βA), but only activin-βA was significantly upregulated [127]. Combined, these experiments underscore the necessary and highly conserved role of TGFβ signalling in spontaneous regeneration, and point towards the activins as potential key players. 

#### 3.2.1. Murphy Roths Large (MRL) Mice

**M**urphy **R**oths **L**arge (MRL/Mpj) mice were originally developed by selective inbreeding for studies of systemic lupus erythematosus, an autoimmune condition with debilitating clinical effects. Surprisingly, however, this mouse strain possesses an exaggerated healing response characterized by the ability to close ear hole wounds and to heal injuries to the myocardium [128,129]. The mechanism behind this increased regenerative ability remains poorly understood, but various lines of evidence point to a role for TGFβ signalling. First, MRL mice demonstrate enhanced levels of the three TGFβ isoforms in various tissues [130], and increased TGFβ response to bacterial infection or **l**ipo**p**oly**s**accharide (LPS) challenge, compared to wild-type mice [131]. Second, two loci strongly correlating to autoimmunity on chromosome 7 and 12 (and possibly responsible for the lupus phenotype in the MRL mice) co-localize with the genes for TGFβ1 and TGFβ3 (respectively) suggesting a possible, albeit speculative, mechanistic link [131,132]. Supporting this possibility, in skin graft models employing MRL mice skin or the skin of a haplotypically identical mouse (B10.BR) on B10.BR recipients suggests that the improved tissue repair in MLR mice is mediated by reduced pro-inflammatory response possibly mediated by TGFβ signalling [133].

#### 3.2.2. TGFβ1 Receptor Mutant Mice

In an attempt to identify candidate genes involved in tissue regeneration, a forward genetics screen using *N*-ethyl-*N*-nitrosurea was used to generate a mouse strain with a fast-healing phenotype identified by ear hole wounding [134]. This phenotype was mapped back to a G to A transition in the gene that codes the TβRI, resulting in a substitution of a conserved arginine residue in the regulatory domain of TβRI. This mutation leads to a modest increase in TGFβ1 responsiveness (two-fold increase as measured by a PAI luciferase vector), as well as a slight increase in SMAD2 phosphorylation [134]. Unfortunately, the responsiveness to other isoforms of TGFβ was not evaluated; however, nearly three-quarters of known TGFβ-responsive genes were not affected by this mutation, thus suggesting tailored modification to the TGFβ signalling pathway. This result demonstrates that receptor-level modifications can lead to phenotypically relevant changes leading to an enhanced regenerative ability, and this situation could be analogous to isoform-specific differences in receptor activation. 

## 4. TGFβ Signalling Targeting in Wound Healing and Tissue Regeneration

As TGFβ signalling drives a number of pathologic conditions, TGFβ-targeting agents have been developed for medical applications in oncology, fibroproliferative disorders, vascular diseases, and wound healing (reviewed in [135]). However, the clinical development of these agents has been challenging, in part due to the fact that TGFβ ligands are highly cell-type and context-dependent. Despite this limitation, the strategies discussed below hold therapeutic promise as potential enhancers of regenerative capacity.

### 4.1. Small Molecule Inhibitors

There are a number of **s**mall **m**olecule **i**nhibitors (SMI) of type II and type I TGFβ receptor kinases, but only the latter have progressed to phase I/II clinical trials (reviewed in [136]). SB-431542, a TβRI SMI discussed above, was extensively used in *in vivo* studies that demonstrated the role of canonical TGFβ signalling in tissue regeneration. However, more specific inhibitors have been developed since. One of these is LY2157299 (Eli Lilly and Company, Indianapolis, IN: Clinicaltrials.gov: NCT01373164), which has progressed to phase II in the oncology setting (reviewed in [137]). Although the application of this and similar SMIs to the improvement of healing and/or regeneration might be limited by their broad inhibition of signalling by different TGFβ family ligands (some of which may be crucial to these processes [123,124]), preclinical studies indicate potential in specific settings. For instance, a study evaluating the role of TGFβ in muscle regeneration found that TGFβ1 serum levels were elevated in older mouse and humans, and this effect was associated with reduced capacity of satellite cells to regenerate muscle in aged individuals [138]. In this study, systemic treatment of older mice with an SMI inhibitor of TβRI ALK4, 5 and 7 (A83-01), but not a neutralizing antibody or decoy receptor, restored the reparative capacity of old muscle [138]. A SMI of TβRI (CAS-446859-33-2) was also observed to improve cardiomyoblast-mediated regeneration in mice [139]. Although little is known of the applicability of TβRI SMIs to improve wound healing, subconjunctival administration of SB-431542 was shown to reduce scar formation after glaucoma surgery in rabbits [140]. As these inhibitors progress through oncological clinical trials, it will be interesting to see how patients fare in the context of post-surgical wound healing following neoadjuvant therapy, as well as overall wound-healing capacity during and after adjuvant treatment.

Another target of SMI are integrins. Previous studies have determined that various integrins (e.g., α_v_β_1_,) mediate non-proteolytic activation of TGFβ1 [141,142]. A SMI of the α_v_β_1_ integrin (c8) has recently been developed, and used to treat two different mouse models of pathologic fibrosis: induced pulmonary fibrosis and induced hepatic fibrosis [142]. Subcutaneous treatment with c8 resulted in a reduction of collagen deposition in both models. The authors concluded that inhibition of α_v_β_1_ integrin by c8 protects against TGFβ1-mediated fibrosis, although other potential integrin-dependent but TGFβ-independent anti-fibrotic mechanisms may also participate [142].

### 4.2. Monoclonal Antibodies 

Compared to SMI, monoclonal antibodies have several distinct advantages, including target ligand specificity and extracellular mode of action. This is particularly relevant to tissue regeneration, as isoform-specific antibodies have the capacity to neutralize specific “inhibitory” ligands in the extracellular space. A number of antibodies directed against TGFβ ligands have progressed through various phases of clinical development [136]. One particularly promising example is fresilimumab (GC-1008, Genzyme/Sanofi, Cambridge, MA, USA), a humanized antibody that targets TGFβ1, TGFβ2 and TGFβ3 ligands. To date, fresilimumab has progressed through phase I clinical trials in focal segmental glomerular sclerosis (NCT00464321), systemic sclerosis (NCT01284322) and idiopathic pulmonary fibrosis (IPF)(NCT0125385) [143].

Isoform-specific monoclonal antibodies against both TGFβ1 (metelimumab, CAT-192) and TGFβ2 (lerdelimumab, CAT-152) have also been developed (Cambridge Antibody Technology, Cambridge, UK; now part of AztraSeneca). Lerdelimumab (targeting TGFβ2) did show promise in glaucoma surgery by reducing scarring during subconjunctival wound healing in a randomized study in rabbits [144]. It also showed promise in a similar phase I/II study, in which the antibody was locally administered (subconjunctival injections) pre- and post-operatively to humans [145]. Although a phase III study that investigated its use in preventing scarring after first-time trabeculectomy for **p**rimary **o**pen-**a**ngle **g**laucoma (POAG) or **c**hronic **a**ngle-**c**losure **g**laucoma (CACG) did not find it beneficial [146], lederlimumab was found to be safe in this and the previously mentioned human trials. Despite discontinued clinical development of both lerdelimumab and metelimumab [147], pre-clinical and clinical studies with these or similar antibodies in different scenarios of healing/regeneration are necessary, as they may still provide the ability for TGFβ isoform-specific modulation of the wound environment in favour of scar-free healing, with potentially minor side effects. 

### 4.3. Ligand Traps/Decoy Receptors

Several TGFβ ligand traps have been developed based on the peptide sequence of the TGFβ co-receptor betaglycan (a TβRIII). One such ligand trap, referred to as P144 or disitertide, is a peptide encompassing amino acids 730−743 from the membrane-proximal ligand-binding domain of betaglycan. P144 acts by interfering with binding and activity of TGFβ1 [148]. Systemic (intraperitoneal) treatment with P144 prevents fibrosis following a chemically induced liver injury in rats [148], while its topical administration ameliorates both bleomycin-induced skin fibrosis in mice [149] and human scar hypertrophy in a xenotransplant model in mice [150]. P144 (disitertide topical cream) is ready to enter phase II clinical trials for potential application in the treatment of localized scleroderma, and phase IIb for systemic sclerosis (http://dignabiotech.com). 

### 4.4. Antisense Oligonucleotides

Another approach to target TGFβ signalling consists of blocking TGFβ ligand gene expression, or the expression of specific SMADs, through the use of **a**nti-**s**ense **o**ligo**n**ucleotides (ASON). These short polymers inhibit target gene expression by binding to target mRNA sequences and blocking mRNA translation. Trabedersen, developed by Antisense Pharma (now Isarna Therapeutics, Munich, Germany), is a TGFβ2-specific ASON with demonstrated efficiency in phase II and III trials in oncology applications, specifically glioblastoma (reviewed by [136]). The evaluation of TGFβ and SMAD-specific oligonucleotides in wound healing and regeneration is still at the preclinical stage, but the results so far are encouraging. Both TGFβ2-targeting and TGFβ1-specific ASONs showed a reduction in post-operative scarring after a single administration at the time of surgery in two different animal models of human glaucoma filtration surgery [151]. In this study, TGFβ2-targeting ASON was determined to be the most effective treatment. A more recent study demonstrated that SMAD3-specific ASON prevents scarring following flexor tendon repair surgery [152]. One advantage of the anti-sense oligonucleotide therapy seems to be a long-lasting effect [151], which might reduce the number of necessary post-surgical administrations. 

### 4.5. Indirect TGFβ-targeting Agents

Anti-TGFβ signalling effects and associated regenerative properties have also been observed in biologically active molecules produced by plants and animals or that were chemically synthesized; some have already been approved for human and veterinary medicine. These include curcumin [153], decorin [154], halofuginone [155], quercetin, asiaticoside, and tetrandine [156]. 

An emerging example is the angiotensin receptor blocker Losartan. In addition to its widespread use in treating hypertension, Losartan also inhibits TGFβ-induced activation of canonical and non-canonical signalling mediators [157]. Related to this, it shows some promise for patients suffering from Marfan syndrome and possibly other inherited connective tissue disorders where excessive TGFβ signalling predisposes to aortic root aneurism and/or skeletal muscle myopathy [157,158]. Losartan treatment at specific doses and schedules also improves muscle healing in a mouse model of contusion-induced muscle injury [159], and facilitates epidermal wound regeneration in a model of streptozotocin-induced diabetes in mice [160].

Another promising compound is **p**ir**f**eni**d**one (PFD), an anti-fibrotic, anti-inflammatory, and antioxidant with demonstrated abilities in down-regulating a number of profibrotic cytokines, including TGFβ1 [161]. PFD has been licensed in many countries (except for the United States) for the treatment of idiopathic pulmonary fibrosis, a chronic lung disease resulting from an aberrant wound-healing circuitry in pulmonary epithelium [162]. PFD nanoparticles, administered 1 h post-injury and daily for up to 7 days, promoted re-epithelization, and decreased collagen type I synthesis and cornea opacity in a mouse model of alkali-induced corneal burn [163]. A more recent study on excisional wound healing in mice tested the effect of PFD delivered using several different topical modalities. Regardless of mode of delivery, PFD was found to accelerate wound contraction and significantly reduce TGFβ expression as well as scarring [164]. 

### 4.6. Recombinant TGFβ

An alternative strategy to the pharmacological approaches described above involves the application of exogenous TGFβ ligands, most notably the recombinant TGFβ3 (Avotermin/Juvista) produced by Renovo (Manchester, UK) [165]. As demonstrated by three randomized, double-blind, placebo-controlled phase II clinical trials (NCT00594581, NCT00432211 and NCT00430326), avotermin treatment is safe, well tolerated, and offers a significant improvement in scar appearance [166,167,168]. Data from *in vitro* and pre-clinical studies (reviewed in [169]) also indicate that avotermin enhances chondrogenesis. Of note is the proposed use of cartilage-ECM-derived scaffolds that might allow for controlled release of TGFβ3 to promote chondrogenesis of intrapatellar fat pad-derived stem cells for use in articular cartilage regeneration [170].

The use of recombinant ligand to promote tissue regeneration might not be limited to TGFβ3. A recent study comparing the effect of TGFβ1 and BMP2 on calvarial defect healing and suture regeneration in rabbits, suggests TGFβ1 to be a superior factor in this particular setting, by promoting bone healing via the native intramembranous ossification pathway [171].

## 5. Conclusions

Both canonical and non-canonical signalling activated by TGFβ isoforms 1, 2 and 3, as well as activin play crucial roles in wound healing and multi-tissue regeneration across vertebrates. The ultimate outcome of this signalling depends on an exquisite balance of ligand levels, the cell type, and the micro-environmental context in which the ligand is presented, including the stiffness of the ECM. In adult mammals, high levels of TGFβ1 and TGFβ2, and low levels of TGFβ3 facilitate scar-forming healing, while in fetal mammals, high levels of TGFβ3 and low levels of TGFβ1 and TGFβ2 favour scar-free healing. ALK-mediated signalling by TGFβ1, TGFβ2 and activin βA drives early stages of blastema-mediated, multi-tissue regeneration in axolotls, *Xenopus*, zebrafish and possibly leopard geckos, with one or more of these ligands playing a prominent role, depending on the species. Canonical signalling by distinct TGFβ isoforms also modulate repair of cardiac and skeletal muscle, bone, and cartilage. Based on the knowledge accumulated over the last three decades, a number of different strategies to modulate TGFβ signalling are either under investigation or have been approved (e.g., recombinant-human TGFβ3) to promote scar-free wound healing and/or regeneration of specific tissues in humans. Further research on regeneration-competent vertebrates is encouraged, as this will lead to the identification of the elements lacking in regeneration-incompetent vertebrates, thus informing pharmacological strategies of broad applicability to both human and veterinary regenerative medicine.

## Abbreviations

The following abbreviations are used in this manuscript:

αSMA: Alpha Smooth Muscle Actin
AER: Apical Ectodermal Ridge
ALK: Activin-Like Kinase
ASON: Anti-Sense Oligonucleotides
BMP: Bone Morphogenic Protein
CACG: Chronic Angle Closure Glaucoma
ECM: Extracellular Matrix
EEA-1: Early Endosome Antigen 1
EMT: Epithelial to Mesenchymal Transition
EndoMT: Endothelial to Mesenchymal Transition
Erk: Extracellular Signal Regulated Kinases
GDF: Growth and Differentiation Factor
JNK: C-Jun N-Terminal Kinases
LPS: Lipopolysaccharide
MAPK: Mitogen-Activated Protein Kinases
MMP: Matrix Metalloproteinase
MRL: Murphy Roths Large
MRTF: Myocardin-Related Transcription Factor
MIS: MullerianInhibiting Substances
PAI: Plasminogen Activator Inactivator
PFD: Pirfenidone
PI3K: Phosphatidylinositide 3 Kinases
PMN: Polymorphonuclear Cells
POAG: Primary Open Angle Glaucoma
SAPK: Stress-Activated Protein Kinases
SMAD: Small Mothers against Decapentaplegic
SMI: Small Molecule Inhibitors
TAZ: Transcriptional Coactivator with PDZ-Binding Motif
TβRI: Transforming Growth Factor Beta Receptor I
TβRII: Transforming Growth Factor Beta Receptor II
TβRIII: Transforming Growth Factor Beta Receptor III
TGFβ: Transforming Growth Factor Beta
VEGF: Vascular Endothelial Growth Factor
YAP: Yes-Associated Protein
